# Correlation between Serum Lipid Levels and Measured Glomerular Filtration Rate in Chinese Patients with Chronic Kidney Disease

**DOI:** 10.1371/journal.pone.0163767

**Published:** 2016-10-03

**Authors:** Yanni Wang, Xilian Qiu, Linsheng Lv, Caixia Wang, Zengchun Ye, Shaomin Li, Qiong Liu, Tanqi Lou, Xun Liu

**Affiliations:** 1 Department of Nephrology, the Third Affiliated Hospital of Sun Yat-sen University, Guangzhou, China; 2 Department of Nephrology, Hainan General Hospital, Haikou, China; 3 Department of Laboratory Medicine, the First Affiliated Hospital of Sun Yat-sen University, Guangzhou, China; 4 Operation Room, the Third Affiliated Hospital of Sun Yat-sen University, Guangzhou, China; 5 School of Software Engineerning, South China University of Tecchonology, Guangzhou, China; Nagoya University, JAPAN

## Abstract

**Introduction:**

Dyslipidemia is often detected in patients with chronic kidney disease (CKD). Previous studies of the relationship between lipid profiles and kidney function have yielded variable results. We aimed to investigate the correlation between serum lipid levels and kidney function evaluated by measured glomerular filtration rate (mGFR) in Chinese patients with CKD.

**Methods:**

A cross-sectional study was conducted on 2036 Chinese CKD patients who had mGFR. Linear regression analysis was performed to evaluate the correlation between different serum lipid levels and mGFR, while logistic regression analysis was used to investigate the association between CKD stages and the risk of different types of dyslipidemia.

**Results:**

The mean age was 55 years and the mean mGFR was 63 mL/min/1.73m^2^. After adjusting for some confounders (age, gender, body mass index, a history of diabetes, fasting glucose, a history of hypertension, systolic blood pressure, diastolic blood pressure, smoking status, hemoglobin, serum potassium, serum albumin, and serum uric acid), serum triglyceride level showed a negative correlation with mGFR (β = -0.006, *P* = 0.006) in linear regression analysis, and CKD stages were positively related to the risk of hypertriglyceridemia (odds ratios were 1.329, 1.868, 2.514 and *P* were 0.046, < 0.001, < 0.001 for CKD stage 2, 3, 4/5, respectively) in logistic regression anlysis.

**Conclusions:**

Serum triglyceride level is independently association with mGFR. Patients with reduced kidney function are more likely to have higher serum triglyceride levels. Further longitudinal, multicenter and well-conducted studies are needed to provide more evidence.

## Introduction

Chronic kidney disease is recognised as a major health problem worldwide. The disease affects 10–16% of the general population in Asia, Europe, and the USA [1–4]and is the risk factor for cardiovascular diseases (CVD)[5]. In patients with impaired kidney function and raised concentrations of albumin in urine, the risk of CVD is two to four times higher than that in individuals with normal kidney function [6].

Dyslipidemia leads to atherosclerosis, and is considered to be an important risk factor for CVD [7,8]. Accelerated atherosclerosis and CVD are the main reasons for death in patients with CKD [9]. Previous studies have shown that total and LDL cholesterol levels usually maintain normal, while triglyceride levels increase and HDL -C levels decrease with the reduced GFR[10–12]. However, the relationship between different types of dyslipidemia and CKD stages remains to be determined. In addition, most previous researches investigating the relationship between lipid profiles and kidney function focused on only certain type of dyslipidemia, such as high-density lipoprotein cholesterol (HDL-C) [13–15]. They usually use the glomerular filtration rate (eGFR) estimated by creatinine and/or cystatin C-based equations to reflect kidney function, which may not be accurate enough[16]. The measured glomerular filtration rate (mGFR) is considered as the gold standard in evaluating kidney function [17]. The current study was designed to investigate the relationship between serum lipid levels (including HDL-C, low-density lipoprotein cholesterol (LDL-C), triglyceride (TG) and total cholesterol (TC)) and mGFR in Chinese patients with CKD.

## Methods

### Patients and Study Design

The cross-sectional study was conducted among Chinese CKD patients with mGFR assessed by a technetium 99m diethylene-triaminepentaacetic acid (^99m^Tc-DTPA) renal dynamic imaging method, from January 2005 to December 2014 at the Third Affiliated Hospital of Sun Yet-sen University, Guangzhou, China. Patients were excluded for any of the following reasons: 1) younger than 18 years old; 2) taking lipid-lowering therapies; 3) with missing data on factors hypothesized to be associated with GFR. 2036 patients were included and staged according to the National Kidney Foundation (NKF) Kidney Disease Outcomes Quality Initiative (K/DOQI) clinical practice guidelines [18]. CKD stage 4 and stage 5 were merged because of their small sample sizes. The study was approved by the institutional review board of the Third Affiliated Hospital of Sun Yat-sen University. We contacted those participants before July 25^th^ 2011 by telephone or letters to get their informed consent, and got approval of ethical review exemption from the medical ethics committee for participants who were unable to contact. Written informed consent was obtained from all the participants after July 25^th^ 2011. The participants specifically consented to participate in this study and to have their medical records used in research.

### Data collection

Information about the age, gender, smoking status, the history of hypertension and diabetes mellitus, and medication use of the participants was collected from their medical records. Weight and height were measured. Body mass index (BMI) was calculated as weight (kg) divided by height squared (m^2^). The blood pressure measurement was performed by well-trained doctors using a calibrated desktop sphygmomanometer (Yuyue, Armamentarium Limited Company, Jiangsu, China) while the participants were seated and rested for at least 5 min.

The measured GFR (mGFR) was obtained by a ^99m^ Tc-DTPA renal dynamic imaging method (modified Gate’s method), using a Millennium TMMPR SPECT with the General Electric Medical System (Discovery VH, GE Healthcare). The details had been described previously [19]. Serum albumin, fasting glucose, serum potassium, serum uric acid, HDL-C, LDL-C, TG and TC were assayed in a single laboratory (Department of Laboratory, The Third Affiliated Hospital of Sun Yet-sun University, Guangzhou, China) on an Hitachi 7180 autoanalyzer (Hitachi reagents from Roche Diagnostics).

All data used in this study was collected as part of standard clinical care. We did not collect any data from patients specifically for this study. The patient data was anonymized before they were accessed by the researchers.

### Definition

Different types of dyslipidemia were defined by the 2007 Guidelines for Prevention and Treatment of Dyslipidemia in Adults in China [20]: (1) hypercholesteremia was defined as TC ≥ 200 mg/dL (5.18 mmol/L); (2) hypertriglyceridemia was defined as TG ≥ 150 mg/dL(1.70mmol/L); (3) hyper LDL cholesteremia was defined as LDL-C ≥ 130 mg/dL (3.37 mmol/L); (4) hypo HDL cholesteremia was defined as HDL-C < 40mg/dL(1.04 mmol/L); (5) dyslipidemia was defined as any of above conditions.

### Statistical Analyses

Continuous variables were presented as the mean and standard deviation (SD), and categorical variables were presented as numbers and percentages. We began with un-adjusted linear regression analysis to evaluate the correlation between different serum lipid levels and mGFR, and models adjusted for age, gender, BMI, a history of diabetes, fasting glucose, a history of hypertension, systolic blood pressure(SBP), diastolic blood pressure (DBP), smoking status, hemoglobin, serum potassium, serum albumin, and serum uric acid. Logistic regression analysis was used to investigate the association between CKD stages and the risk of different types of dyslipidemia: unadjusted models first, and then models adjusted for the same factors mentioned above. *P* value less than 0.05 indicates a significant difference. All statistical analyses were performed using SPSS software (version 16.0; SPSS Inc).

## Results

### Characteristics of participants

Table 1 shows the clinical characteristics of the study participants. A total of 2036 patients were included, of whom 849(41.7%) were female. The study population had an average age of 55 years and mGFR of 63 mL/min/1.73m^2^. There were 1159 (56.9%) participants with a history of hypertension and 939 (46.1%) with a history of diabetes. 289 (14.2%) of them were current smokers. The mean levels of HDL-C, LDL-C, TG and TC were 45.60±23.94, 109.44±47.50, 180.40±168.08 and 186.20±61.18 mg/dL, respectively.

**Table 1:** Characteristics of the study participants. *BMI* indicates body mass index; *SBP* systolic blood pressure; *DBP* diastolic blood pressure; *HDL—C* high-density lipoprotein cholesterol; *LDL-C* low-density lipoprotein cholesterol; *TC* total cholesterol.

Characteristics	
Male (n^a^, %)	1187(58.3)
Age (years)	55.12±15.29
BMI (kg/m2)	23.57±3.68
Hypertension (n, %)	1159 (56.9)
SBP (mmHg)	139.45±23.20
DBP (mmHg)	80.06±14.43
Diabetes (n, %)	939 (46.1)
Fasting glucose (mg/dL)	119.88±61.38
Current smoking(n, %)	289 (14.2)
HDL—C (mg/dL)	45.60±23.94
LDL-C (mg/dL)	109.44±47.50
Triglyceride (mg/dL)	180.40±168.08
TC (mg/dL)	186.20±61.18
Hemoglobin (g/L)	117.41±26.38
Serum potassium (mEq/L)	4.09±0.52
Serum albumin(g/L)	38.42±5.58
Serum uric acid (mg/dL)	7.09±2.49
mGFR (mL/min/1.73 m2)	63.00±30.72

^a^ The number of the population.

### The association between mGFR and levels of different serum lipid components

As shown in Table 2, the levels of HDL-C and total cholesterol decreased as CKD stage progressed (*P* for trend were < 0.001 and 0.013, respectively). We performed linear regression analysis between levels of different serum lipid components and mGFR as continuous variables. The results shown in Table 3 indicated that levels of total cholesterol and HDL-C were positively related to mGFR (r were 0.003 and 0.004, *P* were 0.023 and < 0.001, respectively). However, after adjusting for risk factors, only triglyceride showed a negative correlation with mGFR (β = - 0.006, *P* = 0.006).

**Table 2:** Levels of different serum lipid components and incidences of different types of dyslipidemia in different CKD stages. *HDL—C* indicates high-density lipoprotein cholesterol; *LDL-C* low-density lipoprotein cholesterol; *TC* total cholesterol.

CKD stages	1	2	3	4/5	P for trend
HDL-C(mg/dL)	53.58±31.92	45.98±22.04	42.18±21.66	39.52±14.44	<0.001
LDL-C(mg/dL)	109.44±46.74	113.24±55.48	108.68±45.60	105.26±42.56	0.123
TC(mg/dL)	188.86±50.92	188.86±63.46	185.82±65.36	178.60±60.04	0.013
Triglyceride(mg/dL)	184.80±197.12	176.88±154.88	188.32±180.40	167.20±124.08	0.254
Hypo HDL cholesteremia (n^a^, %)	156 (35.8)	283 (44.5)	320 (52.9)	194 (54.0)	<0.001
Hyper LDL cholesteremia (n, %)	140 (32.1)	195 (30.7)	164 (27.1)	86 (24.0)	0.004
Hypercholesteremia (n, %)	170 (39.0)	236 (37.1)	217 (35.9)	104 (29.0)	0.005
Hypertriglyceridemia (n, %)	197 (45.2)	287 (45.1)	278 (46.0)	166 (46.2)	0.702
Dyslipidemia (n, %)	327 (75.0)	504 (79.2)	490 (81.0)	283 (78.8)	0.106

^a^ The number of the population.

**Table 3:** Linear regression analyses of the association between mGFR and serum lipid components levels. *mGFR* indicates measured glomerular filtration rate; *TC* total cholesterol; *HDL—C* high-density lipoprotein cholesterol; *LDL-C* low-density lipoprotein cholesterol.

	Unadjusted model		Adjusted model^a^	
	r	P	β	P
TC	0.003	0.023	0.003	0.086
Triglyceride	0.001	0.64	-0.006	0.006
HDL-C	0.004	<0.001	0.001	0.128
LDL-C	0.001	0.123	0.001	0.567

^a^ The adjusted model was adjusted for age, gender, body mass index, a history of diabetes, fasting glucose, a history of hypertension, systolic blood pressure, diastolic blood pressure, smoking status, hemoglobin, serum potassium, serum albumin, and serum uric acid.

### The association between CKD stages and different types of dyslipidemia

Table 2 also shows that the incidence of hypo HDL cholesteremia increased as CKD stage progressed (*P* for trend was < 0.001), while the incidence of hyper LDL cholesteremia and hypercholesteremia varied inversely (*P* for trend were 0.004 and 0.005, respectively). Logistic regression model was used to investigate the association between different types of dyslipidemia and CKD stages, and CKD stage 1 was used as reference. In the unadjusted model, CKD stages were positively related to the risk of hypo HDL cholesteremia (odds ratios were 1.439, 2.015, 2.110 and *P* were 0.004, < 0.001, < 0.001 for CKD stage 2, 3, 4/5, respectively). In the multi-variables adjusted model, CKD stages were positively related to the risk of hypertriglyceridemia (odds ratios were 1.329, 1.868, 2.514 and *P* were 0.046, < 0.001, < 0.001 for CKD stage 2, 3, 4/5, respectively). These results are shown in Table 4.

**Table 4:** Logistic regression analysis of the association between CKD stages and different types of dyslipidemia. *CI* indicates confidence intervals.

			Unadjusted model			Adjusted model	
		Crude OR	95% CI	*P*	Adjusted OR ^b^	95% CI	*P*
	CKD 1 ^a^	1	Reference		1	Reference	
Hypercholesteremia	CKD 2	0.923	0.718–1.186	0.532	1.123	0.850–1.484	0.413
	CKD 3	0.875	0.679–1.128	0.304	1.349	0.966–1.884	0.079
	CKD 4/5	0.638	0.474–0.86	0.003	1.433	0.919–2.234	0.112
Hypertriglyceridemia	CKD 2	0.998	0.781–1.274	0.985	1.329	1.004–1.758	0.046
	CKD 3	1.031	0.805–1.321	0.806	1.868	1.340–2.605	<0.001
	CKD 4/5	1.043	0.788–1.381	0.766	2.514	1.632–3.873	<0.001
Hypo HDL cholesteremia	CKD 2	1.439	1.120–1.849	0.004	1.109	0.843–1.458	0.460
	CKD 3	2.015	1.565–2.595	<0.001	1.186	0.860–1.634	0.299
	CKD 4/5	2.110	1.586–2.807	<0.001	0.959	0.633–1.453	0.844
Hyper LDL cholesteremia	CKD 2	0.935	0.719–1.215	0.615	0.975	0.733–1.297	0.860
	CKD 3	0.786	0.601–1.029	0.080	0.889	0.630–1.254	0.504
	CKD 4/5	0.666	0.486–0.913	0.011	1.018	0.645–1.606	0.939
Dyslipidemia	CKD 2	1.273	0.953–1.700	0.102	1.234	0.899–1.692	0.193
	CKD 3	1.420	1.055–1.912	0.021	1.293	0.885–1.889	0.185
	CKD 4/5	1.241	0.889–1.732	0.204	1.237	0.758–2.016	0.395

^a^ used as reference

^b^ Odds ratios(ORs) were adjusted for age, gender, body mass index, a history of diabetes, fasting glucose, a history of hypertension, systolic blood pressure, diastolic blood pressure, smoking status, hemoglobin, serum potassium, serum albumin, and serum uric acid.

## Discussion

Renal dysfunction is usually accompanied by increased levels of triglyceride-rich apoB-containing lipoproteins and decreased levels of apoA-containing lipoproteins[10–12]. In this study, after adjusting for other risk factors, serum triglyceride level showed a negative correlation with mGFR in linear regression analysis, and CKD stages were positively related to the risk of hypertriglyceridemia in logistic regression model. These results were in accordance with the finding in a recent cross-sectional study performed in middle-aged and elderly subjects with normal serum lipid levels in China [21]. In another Chinese community-based cross-sectional survey, researchers drew a conclusion that HDL-C was positively associated with eGFR after adjustment for multiple covariates in the general population [13]. We didn’t come to a similar conclusion in this study. The possible reasons were that lipid components were not used as confounders, and we used mGFR as the outcome instead of eGFR.

The relation between kidney dysfunction and dyslipidemia is still far from fully understood, but some possible pathogenetic connections have recently been revealed. The first mechanism may be altered metabolism of lipoproteins. As shown in kinetic studies of CKD patients with dyslipidemia [22,23], there is a decreased catabolism and elimination of triglyceride-rich apoB-containing lipoproteins, that is due to the impaired lipolysis. On the other hand, the decrease of apoA-containing lipoproteins is caused by a reduction of lipoprotein-A-I [24]. Structural changes and postribosomal modifications of lipoproteins may be the second mechanism. In patients with CKD, the ratio of triglycerides to esterified cholesterol in lipoproteins often changes. There are also postribosomal modifications, such as oxidation, glycation, and carbamilation. Third, insulin resistance, which develops early in kidney dysfunction. One reason is that insulin has a regulatory role in some lipases. Another possible reason is the decreased alpha-type peroxisome proliferator-activated nuclear receptor (PPARa) activity. There are some other mechanisms underlying the dyslipidemia of kidney dysfunction, such as proteinuria and increase of lipoprotein (a) [25].

A major strength of our study is that we used mGFR to evaluate kidney function, which is more accurate than eGFR. Furthermore, this study involved four major types of dyslipidemia and adjusted the regression model by related risk factors. There are some limitations in our study. First, this is a cross-sectional study, which does not reveal the causal relation between serum lipid levels and mGFR. Second, our data represented a specific group of CKD patients in China. Finally, although we adjusted the regression model for 13 covariates that may be associated with mGFR, there are still some other unknown risk factors.

## Conclusions

In conclusion, serum TG level is independently association with mGFR. And further longitudinal, multicenter and well-conducted studies are needed to provide more evidence.

## Supporting Information

S1 STROBE Checklist STROBE_checklist_cross-setional.doc. (DOC)
